# 

**Published:** 1886-07

**Authors:** 


TO OUR SUBSCRIBERS.

The steady and rapid growth of the subscription list and the continued increase of the business interests of the Independent Practitioner make absolutely necessary certain changes in the business management. Heretofore, all of the financial affairs of the journal have been transacted at its New York office, while it was printed and mailed from the editorial office, in Buffalo. It has been found necessary to separate the subscription from the advertising department, and to engage a special clerk to look after the former exclusively. As mistakes have at times unavoidably occurred in the re-transmission of names for the mailing list, it has been thought best to remove the subscription office to Buffalo, and henceforth anything relating to that department, including new names, renewals, removals, and remittances on account of subscriptions, must be sent to that office. The advertising, and all other accounts, will still be kept at the New York office, but as the publishers are all in active practice, it has been found more than any one of them could do to look after both subscriptions and advertising. Remember, then, that henceforth all matters pertaining to subscriptions, as well as editorial communications, should be addressed to the editor, at No. 208 Franklin Street, Buffalo, N. Y.

